# Willingness to Participate and Associated Factors in a Zika Vaccine Trial in Indonesia: A Cross-Sectional Study

**DOI:** 10.3390/v10110648

**Published:** 2018-11-18

**Authors:** Harapan Harapan, Mudatsir Mudatsir, Amanda Yufika, Yusuf Nawawi, Nur Wahyuniati, Samsul Anwar, Fitria Yusri, Novi Haryanti, Nanda Putri Wijayanti, Rizal Rizal, Devi Fitriani, Nurul Fadhliati Maulida, Muhammad Syahriza, Ikram Ikram, Try Purwo Fandoko, Muniati Syahadah, Febrivan Wahyu Asrizal, Kurnia F. Jamil, Yogambigai Rajamoorthy, Abram Luther Wagner, David Alexander Groneberg, Ulrich Kuch, Ruth Müller, R. Tedjo Sasmono, Allison Imrie

**Affiliations:** 1 Medical Research Unit, School of Medicine, Syiah Kuala University, Banda Aceh, Aceh 23111, Indonesia; nur.wahyuniati@unsyiah.ac.id (N.W.); ikramkeren@yahoo.co.id (I.I.); kurnia_jamil@unsyiah.ac.id (K.F.J.); 2 Tropical Disease Centre, School of Medicine, Syiah Kuala University, Banda Aceh, Aceh 23111, Indonesia; 3 Department of Microbiology, School of Medicine, Syiah Kuala University, Banda Aceh, Aceh 23111, Indonesia; 4 School of Biomedical Sciences, University of Western Australia, Nedlands, WA 6009, Australia; allison.imrie@uwa.edu.au; 5 Department of Family Medicine, School of Medicine, Syiah Kuala University, Banda Aceh, Aceh 23111, Indonesia; amandayufika@gmail.com (A.Y.); yusufnawawi@unsyiah.ac.id (Y.N.); 6 Department of Statistics, Faculty of Mathematics and Natural Sciences, Syiah Kuala University, Banda Aceh, Aceh 23111, Indonesia; samsul.anwar@unsyiah.ac.id; 7 School of Medicine, Malikussaleh University, Lhokseumawe, Aceh 24352, Indonesia; fitriayusri1006@gmail.com; 8 Community Health Centre of Meurah Mulia, North Aceh, Aceh 24372, Indonesia; novi.haryanti1985@gmail.com; 9 Community Health Centre of Tapaktuan, South Aceh, Aceh 23711, Indonesia; puput_sbg@yahoo.com; 10 Bunda Hospital, Lhokseumawe, Aceh 24351, Indonesia; dr_rizalmz@yahoo.com; 11 Community Health Centre of Teunom, Aceh Jaya, Aceh 23653, Indonesia; devifitriani.y@gmail.com; 12 Meuraxa Hospital, Banda Aceh, Aceh 23231, Indonesia; nfmaulidaz@gmail.com; 13 Department of Public Health and Community Medicine, School of Medicine, Syiah Kuala University, Banda Aceh, Aceh 23111, Indonesia; syahriza60@gmail.com; 14 Community Health Centre of Gunung, Padang Panjang, West Sumatera 27122, Indonesia; okoex123@gmail.com; 15 Community Health Centre of Lima Kaum, Tanah Datar, West Sumatera 27211, Indonesia; muniatisyahadah94@gmail.com; 16 Community Health Centre of Tanjung Paku, Solok, West Sumatera 27317, Indonesia; febrivanwahyu.asrizal@gmail.com; 17 Department of Internal Medicine, School of Medicine, Syiah Kuala University, Banda Aceh, Aceh 23111, Indonesia; 18 Department of Economics, Faculty of Accountancy and Management, Universiti Tunku Abdul Rahman, Selangor 43000, Malaysia; yogambigai@utar.edu.my; 19 Department of Epidemiology, University of Michigan, Ann Arbor, MI 48109, USA; awag@umich.edu; 20 Institute of Occupational Medicine, Social Medicine and Environmental Medicine, Goethe University, Frankfurt am Main 60323, Germany; groneberg@med.uni-frankfurt.de (D.A.G.); kuch@med.uni-frankfurt.de (U.K.); ruth.mueller@med.uni-frankfurt.de (R.M.); 21 Unit of Medical Entomology, Institute of Tropical Medicine, Antwerp 2000, Belgium; 22 Eijkman Institute for Molecular Biology, Jakarta 10430, Indonesia; sasmono@eijkman.go.id

**Keywords:** Zika, Zika vaccine, vaccine trial, willingness to participate, vaccine acceptance

## Abstract

One of the crucial steps during trials for Zika and other vaccines is to recruit participants and to understand how participants’ attitudes and sociodemographic characteristics affect willingness to participate (WTP). This study was conducted to assess WTP, its explanatory variables, and the impact of financial compensation on WTP in Indonesia. A health facility-based cross-sectional study was conducted in eleven regencies in the Aceh and West Sumatra provinces of Indonesia. Participants were recruited via a convenience sampling method and were interviewed. The associations between explanatory variables and WTP were assessed using a two-step logistic regression analysis. A total of 1,102 parents were approached, and of these 956 (86.8%) completed the interview and were included in analysis. Of those, 144 (15.1%) were willing to participate in a Zika vaccine trial without a financial compensation. In the multivariate analysis, WTP was tied to an age of more than 50 years old, compared to 20–29 years (odds ratio (OR): 5.0; 95% confidence interval (CI): 2.37–10.53), to being female (OR: 2.20; 95% CI: 1.11–4.37), and to having heard about Zika (OR: 2.41; 95% CI: 1.59–3.65). Participants’ WTP increased gradually with higher financial compensation. The rate of WTP increased to 62.3% at the highest offer (US$ 350.4), and those who were still unwilling to participate (37.7%) had a poorer attitude towards childhood vaccination. This study highlights that pre-existing knowledge about Zika and attitudes towards childhood vaccination are important in determining community members being willing to participate in a vaccine trial. Financial incentives are still an important factor to enhance participant recruitment during a vaccine trial.

## 1. Introduction

Zika, caused by Zika virus (ZIKV), is re-emerging public health threat. Reported for the first time in humans in Nigeria in 1954 [1], this disease was subsequently not reported for decades before causing an outbreak in the Yap Islands of the Pacific in 2007 [2]. Since then Zika continued to spread and has been reported in 86 countries in the Americas, Africa, and southeast Asia [3]. Multiple outbreaks of microcephaly cases have also been reported [4,5,6,7], and this devastating complication was one factor that led the World Health Organization (WHO) to declare ZIKV infection as a Public Health Emergency of International Concern (PHEIC) and as an ongoing challenge in 2016. Since then, intense actions have been undertaken to control this new emerging threat, and the development of a vaccine is a key priority [8,9,10,11].

Multiple Zika vaccine candidates are being developed [12,13,14], and one of the key crucial steps before a vaccine can be licensed and approved is that the vaccine should be tested in human clinical trials. One of the challenges in conducting such study is the recruitment of an adequate number of participants. One study found that only 31% of 114 trials in the UK achieved the original recruited target, and 53% of trials required an extension, with difficulties in recruiting a sufficient number of participants as one of the reasons [15]. Consequently, the study may have delayed completion, incur additional costs, or have inconclusive outcomes [15,16,17].

Participant recruitment is dependent on the target populations’ willingness to participate (WTP). Several motivations and barriers have been identified to be associated with WTP in clinical trials [18,19,20]. In Indonesia, only one study has been conducted to assess the WTP in a medical related study and found that being female, working as a civil servant, private employee or entrepreneur, having a high socioeconomic status and good knowledge, attitude and practice related to the disease were associated with WTP [21]. However, new emerging infectious disease with severe complications for infants, such as ZIKV infection, might have additional considerations. It is possible that people may be less aware of the emerging disease or may be more concerned about vaccine safety, and their willingness to be vaccinated may be coloured by their attitudes towards vaccination in general. Understanding how these factors relate to WTP in advance of a clinical trial can lead to better assurance that there will be adequate community participation during the vaccine trial. The current study was conducted to determine the WTP in a Zika vaccine trial, its possible explanatory factors, and the impact of financial compensation on WTP.

## 2. Material and Methods

### 2.1. Study Design and Study Participants

Between 1 February and 13 June 2018, a health facility-based cross-sectional study was conducted in two provinces located on Sumatra in Indonesia, which were Aceh and West Sumatra. The study sites, purposefully selected to include both urban and suburban areas, covered eleven out of 42 regencies or municipalities in those provinces. To recruit study participants, patients who visited outpatient departments of hospitals or community health centres (*Puskesmas*) were recruited via a convenience sampling method and interviewed. Those who were married, have had children or were expecting their first child during the study (collectively called parents) and had resided in the selected regencies for more than 3 months were considered to be eligible.

### 2.2. Study Instrument

A structured questionnaire was designed and developed to assess the WTP and the impact of financial compensation on WTP. The questionnaire was also designed to collect sociodemographic data and other potential explanatory variables of WTP, such as having heard about Zika and support of a Zika vaccine. Attitude towards childhood vaccination, another potential explanatory variable, was also assessed using an established Parent Attitudes about Childhood Vaccines (PACV) questionnaire [22]. The content validity of the questionnaire, including the Indonesian-translated version of PACV, was evaluated by an expert committee consisting of a medical doctor, a family medicine doctor and a microbiologist. The questionnaire was tested for accuracy in a pilot study among ten participants and corrections were made accordingly.

### 2.3. Measures

#### 2.3.1. Dependent Variable

The dependent variable in this study was WTP in a Zika vaccine trial. Prior to the interview, the respondent was informed that: (a) currently, no Zika vaccines have been approved and are available on the market, and they are therefore being developed and tested in trial involving human beings; (b) human trials are important to assess the safety and efficacy of a vaccine prior to its approval to be on the market; (c) the vaccine that is being tested was formulated using an inactivated, subunit, or conjugate vaccine against ZIKV infection and thus could not cause Zika; and (d) there was no information on the adverse effects of vaccine in humans but the vaccine showed no adverse effect during animal study. To ascertain the WTP, with no financial compensation, participants were asked whether they were willing to participate in a vaccine trial for a hypothetical Zika vaccine, as described, where the vaccine needs to be injected intramuscularly and their blood would need to be collected multiple times after vaccination. The possible responses were “yes” or “no”.

#### 2.3.2. Independent Variables

##### a. Sociodemographic Data

We collected sociodemographic data of the participants, including age, gender, educational attainment, employment status, monthly income, and number of children. Educational attainment was defined as the highest level of formal education completed, and employment status was dichotomized into employed and unemployed. Monthly income, the average money earned each month, was measured by asking the participants to choose the closest amount of money from a list in Indonesian Rupiah (IDR), which was then converted to US$ using a July 2018 exchange rate. The number of children was also recorded and grouped into four categories (first pregnancy, 1–2, 3–5, and more than 5 children). We also asked the participants whether they had heard about Zika prior to the present study.

##### b. Attitude for Childhood Vaccination

To measure attitudes for childhood vaccination, the PACV questionnaire was used. The questionnaire measured three sub-domains: vaccination behavior, belief in vaccine safety and efficacy, and general attitude towards childhood vaccination [23]. It consisted of 15 questions with four types of possible responses: “yes”/”no”/”do not know”, 5-point Likert-scale responses (“strongly agree” to “strongly disagree”), 5-point Likert-scale responses (“not at all concerned” to “very concerned”), and a 10-point Likert-scale (“not at all sure” to “completely sure”). As proposed previously [22,23,24], responses to each question were scored as follow: hesitant (scored as 2), not sure (scored as 1), and non-hesitant (scored as 0). Then, the score of each response were summed into an additive scale ranging from 0 to 30, in which higher scores indicate more hesitant for childhood vaccination. For those who were currently expecting their first child during the interview, two questions, within behavior sub-domain were not applicable and therefore the maximum total score was 26. For each respondent, the total raw score of attitudes towards childhood vaccination and its sub-domain was converted into a 0–100 scale and then dichotomized into two categories: good attitude (score < 50) and poor attitude (score ≥ 50), as recommended [23,24,25,26].

##### c. Support to Zika Vaccine

Before assessing acceptance of the vaccine, brief information about Zika was provided to participants. Participants were told that the Zika vaccine was safe, 90% protective vaccine against ZIKV, and needs to be injected into the body through the deltoid muscle. Acceptance of a hypothetical Zika vaccine was assessed by asking the likelihood of participants: (a) to be vaccinated and (b) to recommend their partner to get vaccinated against ZIKV infection. The possible responses were on a five-point Likert-type scale (from “very unlikely” to “very likely”) and scored as follow: 0 was given for “very unlikely” and “unlikely”, 1 for “undecided” and 2 for “likely” and “very likely”. The two questions were added together, with a score ranging from 0 to 4, which was further dichotomized into “willing” and “not willing” based on a 75% cut-off point (i.e. score 3 or more classified as “willing”).

##### d. The Role of Financial Compensation

To assess the impact of financial compensation on WTP, the participants were asked whether they were willing to participate based on varying amounts of money as compensation. The bids were selected randomly from a list (US$ 3.5, 7.0, 17.5, 35.0, 52.6, 70.1, 140.1, and 350.4). The possible responses were “yes” or “no”.

### 2.4. Statistical Analysis

Based on the conservative assumption that the WTP rate was 50% with a confidence interval of 95% and a 5% margin of error, the minimum sample size required was 385. The possible explanatory variables influencing WTP in a Zika vaccine trial were explored using a two-step logistic regression analysis. All explanatory variables (sociodemographic, attitude towards childhood vaccination and support of vaccine) were included in the univariate analysis. Explanatory factors with *p* ≤ 0.25 in this step were entered into the multivariate analysis. All estimated odds ratio (OR) was interpreted in relation to a reference category (R). The possible confounding factors were assessed by comparing the crude OR and the adjusted OR (aOR) [27,28,29,30,31,32]. All analyses were performed using SPSS for Windows (Version 15, Chicago, IL, USA).

### 2.5. Ethical Consideration

In compliance with national legislations and the code of ethical principles in the Declaration of Helsinki, the protocol of this study was approved by the Institutional Review Board of the School of Medicine, Syiah Kuala University, Banda Aceh, Indonesia (19/EA/FK/2018). Prior to the interview, the participants were: (a) provided with a brief information of the study aims, risks and benefits; (b) informed that participation in this study was voluntary and no incentive will be given; (c) informed that they could withdraw any time during the interview; and (d) asked to sign a pre-coded informed consent form once they agreed to participate. To ensure the anonymity of the respondent and confidentiality of the data, an identifier code was assigned for each questionnaire and its matching informed consent form and this code was used in all analyses.

## 3. Results

### 3.1. Participants’ Characteristics

Out of 1102 participants who were contacted, a total of 956 (86.8%) were included in the analysis; data from 145 (13.2%) participants were excluded due to incomplete interviews. A majority (86.5%) of the participants were female and approximately half were between 30–39 years old (Table 1). Approximately 45% of respondents had attended a university, and none were illiterate. There was approximately an equal proportion of employed and unemployed participants (50.7% vs. 49.3%). More than half of the participants earned less than IDR 3 million (US$ 210.2) every month and approximately 60% of them had 1–2 children. In addition, this study found that only 252 (26.4%) of participants had heard about Zika prior to the interview. Overall, 84.1% of participants had a good attitude towards childhood vaccination and the prevalence of participants who had good scores for each sub-domain was 60.2% for vaccination behavior, 92.8% for general attitude towards vaccination, and 38.4% for vaccine safety and efficacy. In total, 79.2% of participants stated their support for Zika vaccine.

### 3.2. Factors Associated with Willingness to Participate

In this study, only 144 (15.1%) participants were willing to participate in a Zika vaccine trial without financial compensation. The univariate analysis found that age more than 50 years old, being female, having heard about Zika and supporting Zika vaccine were all associated with WTP (*p* < 0.05) (Table 2). Education, employment status, monthly income, number of children, attitude towards childhood vaccination were all not associated with WTP.

After excluding explanatory variables with *p* > 0.25, our multivariate analysis revealed that an age of more than 50 years old, being female, and having heard about Zika were the only variables associated with WTP. Participants in the oldest age group (more than 50 years old) had five times higher odds of WTP compared to those between 20–29 years (OR: 5.00; 95% confidence interval (CI): 2.37–10.53). Females had double the odds of being willing to participle in a Zika vaccine trial compared to males (OR: 2.20; 95% CI: 1.11–4.37). Additionally, having heard about Zika was significantly associated with WTP in a Zika vaccine trial (OR: 2.41; 95% CI: 1.59–3.65). Attitudes towards childhood vaccination and its subdomains had no association with higher WTP in a vaccine trial.

### 3.3. Financial Compensation and Willingness to Participate

**Figure 1:**
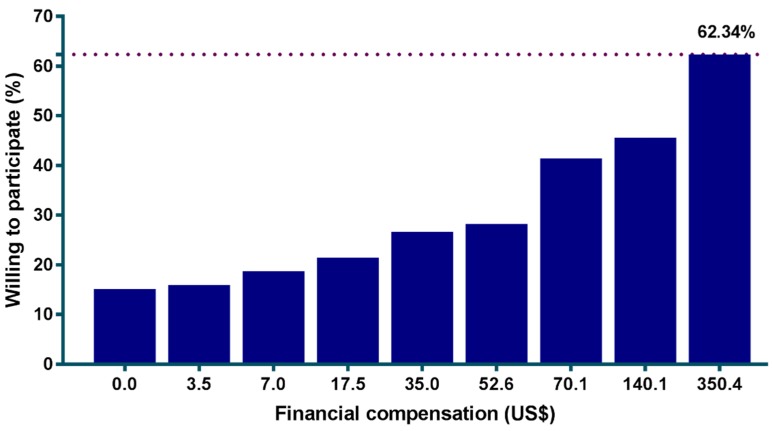
Relationship between financial compensation and the proportion of participants who are willing to participate in a Zika vaccine trial in Aceh and West Sumatra, Indonesia. Purple dotted line indicates the maximum percentage of participants who are willing to participate at the highest financial compensation.

Financial compensation increased WTP among participants. Without financial compensation, 15.1% of participants were willing to participate; this percentage increased gradually with a higher offer of financial compensation, from 18.7% for US$ 7.0 to 26.6% for US$ 35.0. The percentage of participants who were willing to participate increased up to 62.3% (596/956) at the highest financial compensation offered, US$ 350.4 (equal to IDR 5 million). There were 360 (37.7%) of participants who persisted in being unwilling to participate in vaccine trial even at the highest offered compensation (Figure 1). Our stratified analysis using logistic regression found that unwillingness to participate even with financial compensation was associated with a poor attitude towards childhood vaccination (OR: 1.67; 95% CI: 1.17–2.36, *p* = 0.001).

## 4. Discussion

To the best of our knowledge, this is the first study reporting the WTP and its associated factors in the context of a Zika vaccine trial. Our study found that WTP in a Zika vaccine trial was very low (15.1%) in Indonesia, and that age, gender and having heard about Zika were significantly associated with WTP. Researchers looking to recruit participants into a vaccine trial could consider these factors before starting a trial to enhance participation, minimize recruitment costs, and maintain generalizability of the study population.

Our study identified that being female was associated with a higher WTP. This could be related to a previous study’s findings, which showed that women are more altruistic than men in Indonesia [33]. Many previous studies have identified altruism, acting with an unselfish regard for others, as one of the most important factors for WTP in clinical trials or other medical studies [34,35,36,37,38,39,40,41]. One study specifically identified that women were more likely to participate in a trial because of general altruistic considerations [39]. In Indonesia, a previous study similarly found that women were more willing to donate their blood for dengue research [21]. Together, our study on Zika and the previous study on Dengue [21] indicate that females are likely more willing to participate in health-related research. However, this finding should also be interpreted carefully because the Zika vaccine trial can prevent complications in pregnancy, and so women may inherently be more attuned to the benefits of the trial’s outcome. In Indonesia, a wife usually consults with her partner on issues related to the household, especially in regards to pregnancy [42]. The husband, in turn, has an important role in influencing the behaviour of pregnant women [42] and wives traditionally put their husband’s and father’s opinion before their own [43]. Similar societal characteristics are found in other low- and middle-income countries where women’s decision to participate was frequently influenced by their partner or family [39,44]. In contrast, this phenomenon is not observed in high-income countries such as the US [45]. In addition, friends and health professionals are other important influencers related to WTP [44]. These findings indicate that the decision making process to participate in a clinical trial or another medical-related study is complex and influenced by core family members and other community members. In the context of a Zika vaccine program, we believe that the decision may be more complex because of Zika’s association with pregnancy complication, which may then require additional communication between wife and husband. Therefore, information related to Zika vaccine trials should be provided not only to study participants, but should also be tailored and made available to other core family members, if the study participant asks for it.

Another influencing factor for WTP that was identified in this study was having heard about Zika prior to the study. We assumed that participants who had heard about Zika had better pre-existing knowledge about Zika, which would promote WTP. Additionally, good knowledge about Zika produced good perception to the Zika disease. Previously, studies clearly indicated that good knowledge [21] and good perception of disease risk [46] were predictors for a high WTP. Previous studies have also found that community members who have good knowledge regarding a medical study [47], good perception and high awareness concerning medical research [48], and prior participation in clinical trials [49] are more likely to have a positive response to WTP. In contrast, the lack of knowledge and awareness about a medical study is a significant barrier to participating [48,50]. These studies highlight the importance of not only adequately providing information about Zika to potential participants, but also explaining the utility of clinical trials in general in order to increase positive perceptions about participating in studies.

Our study also indicated that offering a financial incentive increased participants’ WTP. By providing financial compensation up to US$ 350, the percentage of WTP increased by 47.2% compared to no compensation (62.3% vs. 15.1%). The increased rate in our study is higher compared to another study that found a 12.2% absolute increase in those reporting that they would not participate after mentioning financial incentive [49]. This difference between studies could be influenced by the amount of the money offered in the study. It has been previously observed that financial compensation is positively associated with WTP in vaccine trials [49,51].

There are concerns about the use of financial incentives [52,53]—for instance, that the use of money can remove the voluntary aspect of participating in trials, by being unduly coercive; but evidence suggests that, at least in high-income countries, greater compensation is not associated with participants being willing to undergo riskier procedures [54]. Additionally, compensation can reduce financial barriers for participants, overcome opportunity costs, inertia, and distrust, and fairly compensate for the time and inconvenience that participants incur [52].

Interestingly, although incentive compensation increased the proportion of WTP, more than a third of the participants (37.7%) were persistently unwilling to participate even with financial compensation. Initially we hypothesized that this group might be the wealthiest group of participants. However, our stratified analysis indicated that this unwillingness to participate was associated with poor attitude towards childhood vaccination and had no association with monthly income. Solid evidence from previous studies also revealed that good attitude towards the disease or clinical studies were one of the strongest predictors for WTP [21,48,55,56]. A study also found that a good attitude towards childhood vaccination was the strongest predictor for acceptance for a hypothetical Zika vaccine [57]. This underscores that basic attitude towards the disease and vaccination are critical cornerstones that influence participant recruitment into a vaccine clinical trial.

In this study we have identified factors associated with WTP in a Zika vaccine trial and have suggested some efforts to be undertaken. Those efforts can mitigate well-known barriers of WTP such as safety concerns, worries about vaccine-induced seropositivity, side effects, mistrust of researchers, and concerns about trial duration, visit frequency and travel distance [51,58,59,60]. We are concerned that the magnitude of individuals who were not willing to participate in a clinical trial could affect generalizability of future clinical trials in Indonesia. An older study population skewed towards one gender may have different biological responses to a vaccine candidate. Providing financial compensation and educating the community about the benefits of clinical trials and of vaccination are some ways to increase the response rate.

This study had some limitations. The measured WTP was only a stated behavioral intention and therefore may not reflect or predict actual enrolment of community members into a Zika vaccine trial in Indonesia. We did not assess trust, altruism, and psychological barriers which could affect actual participation rates and which may mediate some associations we did observe in this study. We are also unable to confirm the role knowledge of Zika on WTP. Finally, the sociocultural background of participants from the two provinces in this study may not be representative of Indonesia as a whole because of this country’s diversity. By undertaking a convenience sample from health facilities, we may have also biased our sample towards individuals who were sicker or who were more likely to receive medical treatment, which could have both affected WTP in a clinical trial.

## 5. Conclusion

Our study found that the WTP in a Zika vaccine study was low among community members in Indonesia and that being female and knowing about Zika prior to the study were associated with higher WTP. Efforts to increase knowledge and general attitudes towards vaccination among community members may therefore be critical to enhance participant recruitment. Financial incentives are also important to increase the participation rate of community members and to maintain a generalizable study population.

## Figures and Tables

**Table 1:** Participants’ characteristics (*n* = 956).

Variable	*n* (%)
Age group (years)	
20–29 (*R*)	230 (24.1)
30–39	469 (49.1)
40–49	189 (19.8)
More than 50	68 (7.1)
Gender	
Male (*R*)	129 (13.5)
Female	827 (86.5)
Educational attainment	
Primary school (*R*)	42 (4.4)
Junior high school	106 (11.1)
Senior high school	361 (37.8)
Diploma certificate	223 (23.3)
University graduate	224 (23.4)
Employment status	
Unemployment (*R*)	471 (49.3)
Employee	485 (50.7)
Types of workplace	
Non–healthcare sector (*R*)	260 (53.6)
Healthcare sector	225 (46.4)
Monthly income (IDR)	
Less than 3 million (*R*)	523 (54.7)
3–5 million	328 (34.3)
More than 5 million	105 (10.9)
Number of children	
The first pregnancy (*R*)	31 (3.2)
1–2	564 (59.0)
3–5	337 (35.3)
More than 5	24 (2.5)
Have heard about Zika	
No (*R*)	704 (73.6)
Yes	252 (26.4)
Attitude towards childhood vaccination	
Poor (*R*)	152 (15.9)
Good	804 (84.1)
Vaccination behaviour sub-domain	
Poor (*R*)	368 (39.8)
Good	557 (60.2)
General attitude sub-domain	
Poor (*R*)	69 (7.2)
Good	887 (92.8)
Vaccine safety and efficacy sub-domain	
Poor (*R*)	589 (61.6)
Good	367 (38.4)
Acceptance for Zika Vaccine	
Unwilling (*R*)	199 (20.8)
Willing	757 (79.2)

**Table 2:** Univariate and multivariate logistic regression analysis showing predictors of willingness to participate (the second project) in a Zika Vaccine Trial (Willing vs. Not willing) (*n* = 956).

Variable	Willing to ParticipateYes/No	Univariate		Multivariate	
		OR (95% CI)	*p*-Value	aOR (95% CI)	*p*-Value
Age group (years)					
20–29 (*R*)	32/198	1		1	
30–39	61/408	0.93 (0.58–1.47)	0.740	1.29 (0.77–2.15)	0.328
40–49	30/159	1.17 (0.68–2.00)	0.574	1.83 (0.97–3.44)	0.061
More than 50	21/47	2.77 (1.46–5.22)	0.002	5.00 (2.37–10.53)	<0.001
Gender					
Male (*R*)	11/118	1		1	
Female	133/694	2.06 (1.08–3.92)	0.029	2.20 (1.11–4.37)	0.025
Educational attainment					
Primary school (*R*)	4/38	1		1	
Junior high school	24/82	2.78 (0.90–8.58)	0.075	2.88 (0.90–9.25)	0.075
Senior high school	56/305	1.74 (0.60–5.08)	0.308	1.95 (0.64–5.97)	0.240
Diploma certificate	23/200	1.09 (0.36–3.34)	0.877	1.14 (0.34–3.83)	0.827
University graduate	37/187	1.88 (0.63–5.59)	0.256	1.84 (0.56–6.05)	0.317
Employment status					
Unemployment (*R*)	79/392	1		1	
Employee	65/420	0.77 (0.54–1.10)	0.146	0.78 (0.49–1.26)	0.313
Types of workplace					
Non–healthcare sector(*R*)	34/226	1		–	
Healthcare sector	31/194	1.06 (0.63–1.79)	0.821		
Monthly income (IDR)					
Less than 3 million (*R*)	77/446	1		–	
3–5 million	53/275	1.12 (0.76–1.63)	0.571		
More than 5 million	14/91	0.89 (0.48–1.64)	0.712		
Number of children					
The first pregnancy (*R*)	7/24	1		1	
1–2	87/477	0.63 (0.26–1.50)	0.292	0.46 (0.18–1.18)	0.107
3–5	45/292	0.53 (0.22–1.30)	0.164	0.28 (0.10–0.79)	0.016
More than 5	5/19	0.90 (0.25–3.30)	0.876	0.38 (0.09–1.60)	0.187
Have heard about Zika					
No (*R*)	86/618	1		1	
Yes	58/194	2.15 (1.48–3.11)	<0.001	2.41 (1.59–3.65)	<0.001
Attitude towards childhood vaccination					
Poor (*R*)	18/134	1		1	
Good	126/678	1.38 (0.82–2.35)	0.228	1.08 (0.60–1.93)	0.802
Vaccination behaviour sub-domain					
Poor (*R*)	44/324	1			
Good	93/464	1.48 (1.00–2.17)	0.048	–	
General attitude sub-domain					
Poor (*R*)	9/60	1			
Good	135/752	1.20 (0.58–2.47)	0.627	–	
Vaccine safety and efficacy sub-domain					
Poor (*R*)	87/502	1			
Good	57/310	1.06 (0.74–1.53)	0.749	–	
Acceptance for Zika Vaccine					
Unwilling (*R*)	20/179	1		1	
Willing	124/633	1.75 (1.06–2.89)	0.028	1.53 (0.88–2.67)	0.128

aOR: Adjusted odds ratio; CI: confidence interval; OR: odds ratio; *R*: reference group.
